# Nanoparticle enhanced MRI can monitor macrophage response to CD47 mAb immunotherapy in osteosarcoma

**DOI:** 10.1038/s41419-018-1285-3

**Published:** 2019-01-15

**Authors:** Suchismita Mohanty, Ketan Yerneni, Johanna Lena Theruvath, Claus Moritz Graef, Hossein Nejadnik, Olga Lenkov, Laura Pisani, Jarrett Rosenberg, Siddhartha Mitra, Alejandro Sweet Cordero, Samuel Cheshier, Heike E. Daldrup-Link

**Affiliations:** 1 Department of Radiology, Molecular Imaging Program at Stanford, Stanford University, Stanford, CA 94305 USA; 2 Department of Pediatrics, Stanford University, Stanford, CA 94305 USA; 3 Department of Neurosurgery, Institute for Stem Cell Biology and Regenerative Medicine and Division of Pediatric Neurosurgery, Lucile Packard Children’s Hospital, Stanford University, Stanford, CA 94305 USA; 4 Department of Pediatrics, Morgan Adams Foundation Pediatric Brain Tumor Research Program, Children’s Hospital Colorado, University of Colorado Anschutz Medical Campus, Aurora, CO USA; 5 Department of Pediatrics, University of California, San Francisco, San Francisco, CA 94143 USA; 6 Huntsman Cancer Institute, Department of Neurosurgery, Division of Pediatric Neurosurgery, University of Utah, Salt Lake City, UT 84132 USA

## Abstract

CD47 monoclonal antibodies (mAbs) activate tumor-associated macrophages (TAMs) in sarcomas to phagocytose and eliminate cancer cells. Though CD47 mAbs have entered clinical trials, diagnostic tests for monitoring therapy response in vivo are currently lacking. Ferumoxytol is an FDA-approved iron supplement which can be used “off label” as a contrast agent: the nanoparticle-based drug is phagocytosed by TAM and can be detected with magnetic resonance imaging (MRI). We evaluated if ferumoxytol-enhanced MRI can monitor TAM response to CD47 mAb therapy in osteosarcomas. Forty-eight osteosarcoma-bearing mice were treated with CD47 mAb or control IgG and underwent pre- and post-treatment ferumoxytol-MRI scans. Tumor enhancement, quantified as T2 relaxation times, was compared with the quantity of TAMs as determined by immunofluorescence microscopy and flow cytometry. Quantitative data were compared between experimental groups using exact two-sided Wilcoxon rank-sum tests. Compared to IgG-treated controls, CD47 mAb-treated tumors demonstrated significantly shortened T2 relaxation times on ferumoxytol-MRI scans (*p* < 0.01) and significantly increased F4/80+CD80+ M1 macrophages on histopathology (*p* < 0.01). CD47 mAb-treated F4/80+ macrophages demonstrated significantly augmented phagocytosis of ferumoxytol nanoparticles (*p* < 0.01). Thus, we conclude that ferumoxytol-MRI can detect TAM response to CD47 mAb in mouse models of osteosarcoma. The ferumoxytol-MRI imaging test could be immediately applied to monitor CD47 mAb therapies in clinical trials.

## Introduction

Osteosarcoma is the most common primary bone cancer in children and young adults^1,2^. Despite advances in diagnosis, surgery, and chemotherapy, patients with metastasized osteosarcoma have a poor prognosis, with a 2-year event-free survival rate of 15 to 20%^3^. The transmembrane protein CD47 represents a promising new target for the treatment of osteosarcoma^4^. CD47 is a cell-surface molecule on osteosarcoma cells that function as a “don’t eat me” signal by engaging signal-regulatory protein alpha (SIRPα), an inhibitory receptor on macrophages^5,6^. CD47 monoclonal antibodies (mAbs) inhibit the interaction between CD47 and SIRPα, and thereby activate tumor-associated macrophages (TAMs) to phagocytize cancer cells^7–11^. However, the complex heterogeneity of osteosarcomas can lead to variable responses to immunotherapies and emergence of resistant tumor subtypes. As CD47 mAb therapies are entering clinical trials, minimally invasive imaging methods that can monitor therapy response longitudinally in vivo are urgently needed.

Previous investigations have shown that ferumoxytol nanoparticles are phagocytosed by TAMs and can be detected with magnetic resonance imaging (MRI) in mouse models^12^ and patients^13^. Ferumoxytol is approved by the Food and Drug Administration (FDA) as an iron supplement and can be used “off label” as a contrast agent for MRI^14–17^. It is not known if ferumoxytol-MRI can monitor tumor response to CD47 mAb therapy. Since CD47 mAbs activate TAM phagocytosis in tumors, we hypothesized that successful CD47 mAb therapy would increase ferumoxytol nanoparticle retention and T2-signal enhancement of the tumor tissue. Therefore, the purpose of our study was to evaluate if ferumoxytol-MRI can monitor TAM response to CD47 mAb therapy in osteosarcomas. This imaging test could immediately inform ongoing clinical trials with CD47 mAb.

## Results

### Presence of CD47 and TAMs in human osteosarcoma

**Fig. 1:**
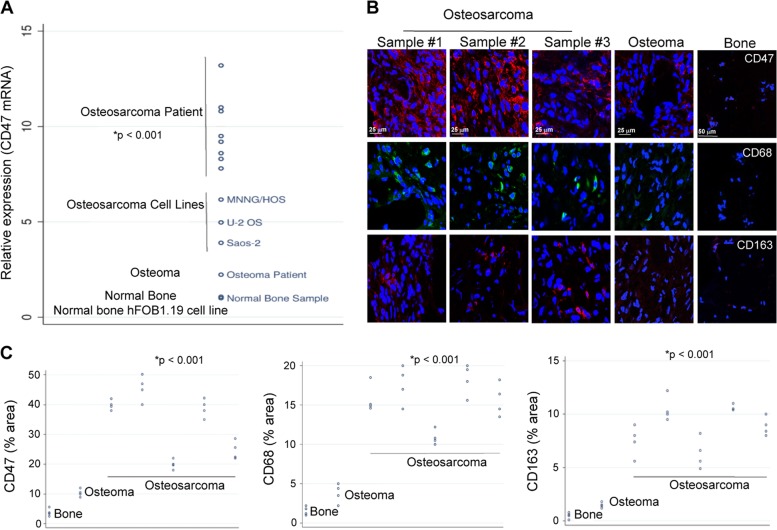
CD47 and tumor-associated macrophages (TAMs) are abundant in osteosarcoma patient specimens. **a** CD47 gene expression in a normal bone cell line, a normal patient bone, and osteoma patient specimen, three different osteosarcoma cell lines and eight osteosarcoma patient specimens. CD47 expression was measured with quantitative real-time PCR (qPCR), using glyceraldehyde 3-phosphate dehydrogenase (GAPDH) as an endogenous control, and compared between osteosarcomas and osteomas and normal bone using exact two-sided Wilcoxon rank-sum tests, *p* < 0.001. **b** Representative confocal CD47, CD68, and CD163 stains of three human osteosarcoma samples (scale bar 25 μm), a human osteoma sample (scale bar 25 μm), and a human bone sample (scale bar 50 μm) as negative control. **c** Corresponding quantitative area of CD47, CD68, and CD163 stains of one bone, one osteoma, and five osteosarcoma samples was measured over four high-power fields with Image J. Differences in CD47-, CD68-, and CD163-stained area of osteosarcoma, osteoma, and bone specimens were tested with exact two-sided Wilcoxon rank-sum tests, *p* < 0.001

As a prerequisite for planned immunotherapies with CD47 blocking antibodies, we first confirmed that human osteosarcomas express CD47. We found that *CD47* messenger RNA (mRNA) expression, as determined by quantitative real-time PCR (qPCR), was significantly higher in human osteosarcoma specimen as compared to osteoma and normal bone specimen (*p* < 0.001; Fig. 1a). Immunofluorescence staining confirmed that CD47 protein levels of all osteosarcoma patient specimens were significantly (*p* < 0.001) higher compared to osteomas and normal bone (Fig. 1b, c). CD47-expressing osteosarcoma specimens showed significantly (*p* < 0.001) higher levels of TAM markers CD68 and CD163 compared to osteomas and normal bones (Fig. 1b, c). We also confirmed the expression of CD47 in MNNG/HOS, Saos-2, U-2 OS, and K7M2 cell lines, respectively, using qPCR and immunofluorescence staining (Fig. 1a, Supplementary Fig. S1).

### CD47 mAb activate phagocytic activity of macrophages in vitro

**Fig. 2:**
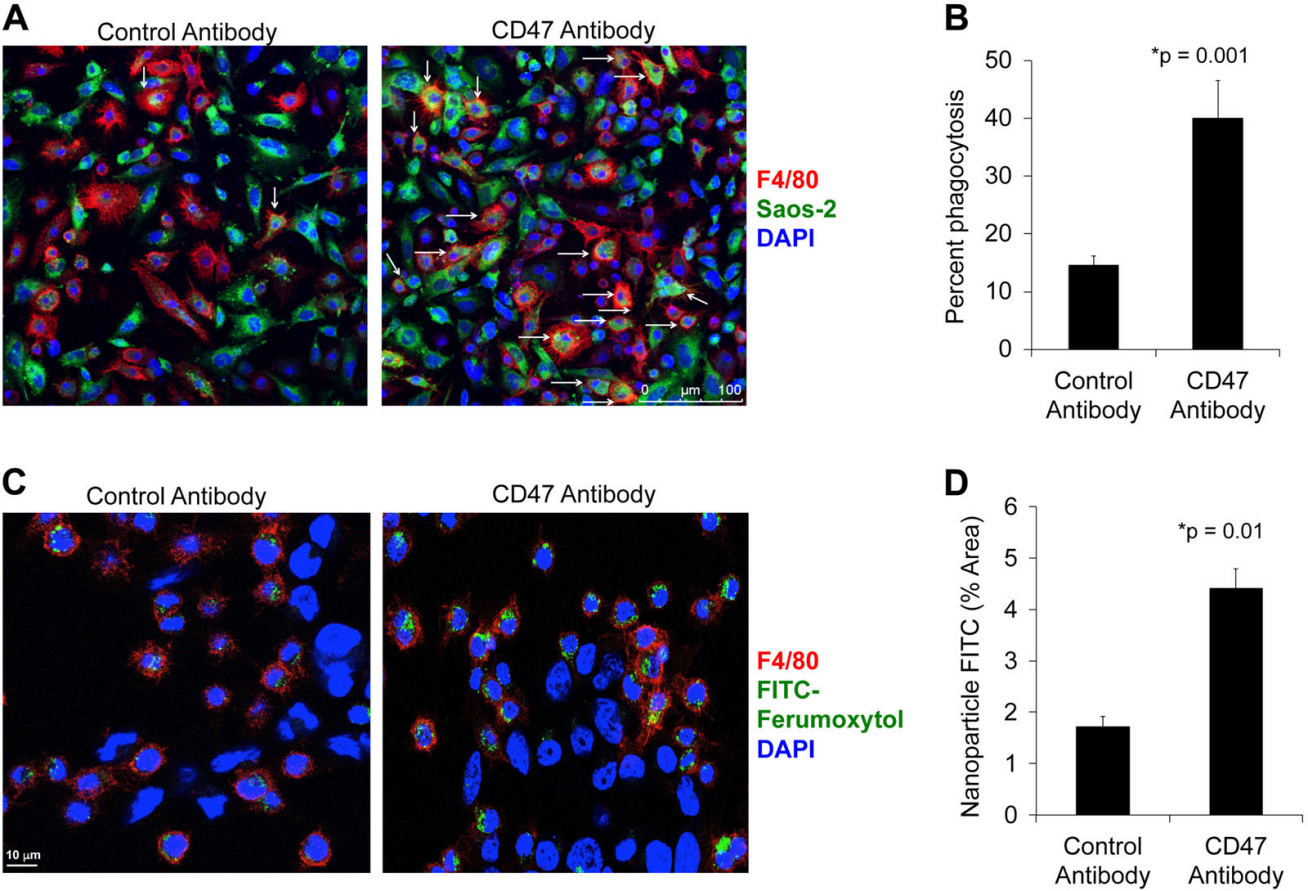
CD47 inhibition triggers tumor cell and nanoparticle phagocytosis in vitro. **a** Confocal images of CellBrite green-labeled Saos-2 tumor cells and F4/80^+^ murine macrophages in the presence of control IgG monoclonal antibody (mAb) (left) and CD47 mAbs (right; 10 μg/mL). CD47 mAb-exposed samples show an increased quantity of phagocytized tumor cells in macrophages (arrows; scale bar 100 μm). **b** Corresponding relative phagocytosis, calculated as the number of macrophages with phagocytized cancer cell divided by total macrophages per five high-power field × 100%. Data are displayed as means ± SD of *n* = 5 experiments per group, *p* = 0.001, exact two-sided Wilcoxon rank-sum tests. **c** Co-culture of F4/80^+^ macrophages, unlabeled MNNG/HOS cancer cells, and fluorescein isothiocyanate (FITC)-loaded ferumoxytol nanoparticles (scale bar 10 μm) in the presence of IgG control mAb or CD47 mAb. **d** Corresponding quantitative area of FITC stains in F4/80^+^ macrophages in control and CD47 mAb-treated samples for five high-power fields was measured using ImageJ. Results are represented as mean ± SD from five independent experiments, *p* = 0.01, exact two-sided Wilcoxon rank-sum tests

We investigated with confocal microscopy whether treatment with CD47 mAb activates the phagocytic activity of macrophages in vitro, in co-cultures with Saos-2 osteosarcoma cells. In the presence of CD47 mAb, F4/80+ macrophages displayed a three- to five-fold increase in phagocytosis of Saos-2 osteosarcoma cells relative to control antibody-treated macrophages (Fig. 2a, b). Furthermore, co-cultures of F4/80+ macrophages, MNNG/HOS osteosarcoma cells and CD47 mAb lead to significantly increased ferumoxytol nanoparticle phagocytosis by macrophages (*p* = 0.01) as compared to co-cultures with control antibody (Fig. 2c, d).

**Fig. 3:**
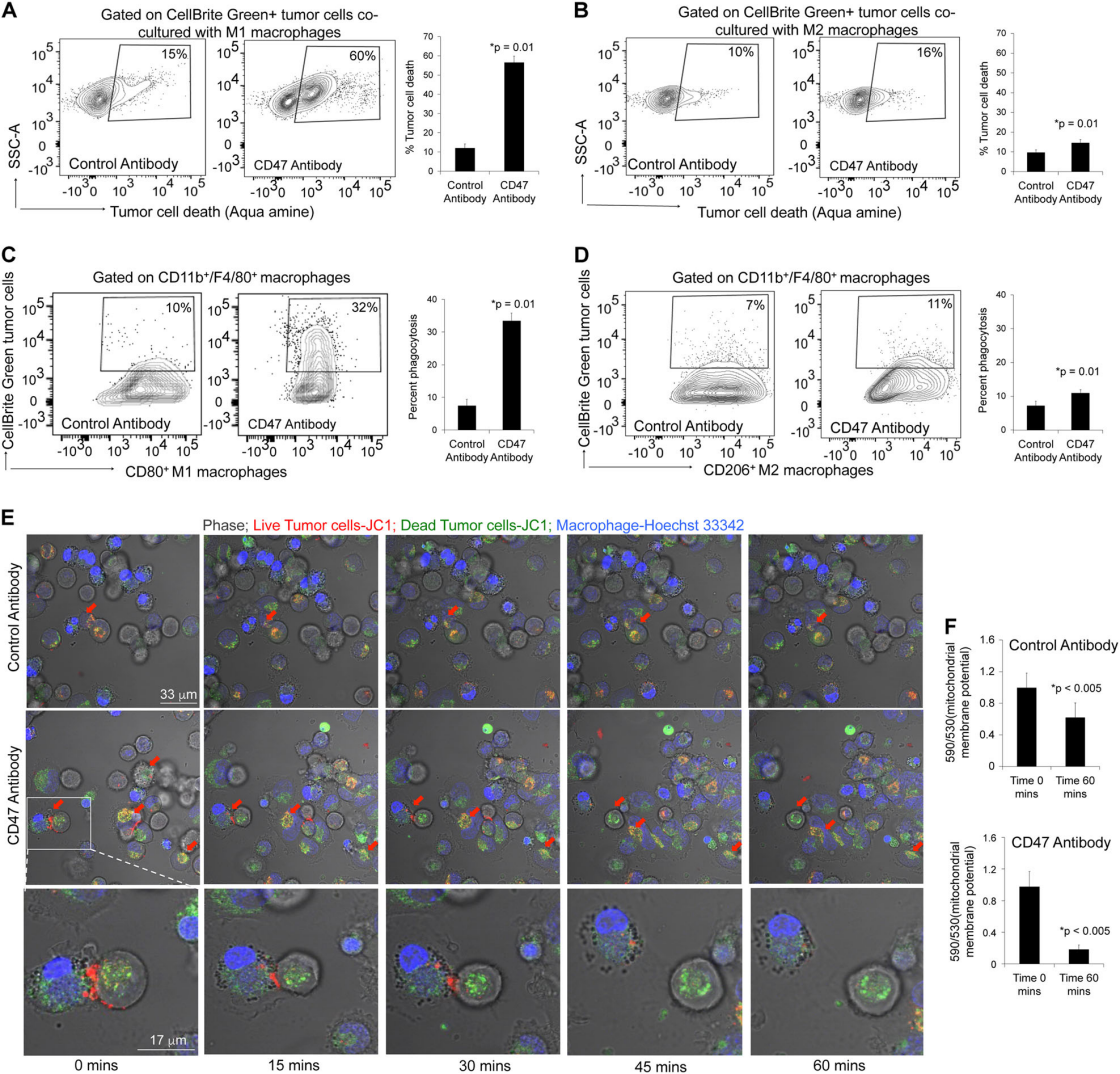
Tumoricidal effects of M1 macrophages in the presence of CD47 monoclonal antibody (mAb). Flow cytometry contour plots and corresponding charts showing tumor cell death when co-cultured with **a** M1 macrophages and **b** M2 macrophages in the presence of CD47 and control mAb. Flow cytometry contour plots and corresponding charts showing phagocytosis of CellBrite green-labeled K7M2 tumor cells by **c** M1 macrophages and **d** M2 macrophages in the presence of CD47 and control mAb. Results are represented as mean ± SD from five independent experiments. **e** Time-lapse confocal images to illustrate tumor cell death during phagocytosis when co-cultured with M1 macrophages in the presence of CD47 and control mAb. Red arrow indicates phagocytosis. **f** Charts representing loss of mitochondrial membrane potential in tumor cells during M1-mediated tumor phagocytosis macrophages in the presence of CD47 and control mAb. Results are represented as mean ± SD from three independent experiments, *p* value as indicated, exact two-sided Wilcoxon rank-sum tests

Using flow cytometry we further investigated whether CD47 mAb treatment causes cancer cell death in vitro. The gating strategy for identifying tumor and macrophages in co-culture experiments is outlined in Supplementary Fig. S2. We found that MNNG/HOS tumor cells underwent significant loss of cell viability in the presence of macrophages and CD47 mAb (60%) when compared to co-cultures treated with control mAb (15%) (Fig. 3a). Further, we observed that the tumoricidal effects of M1 macrophages were significantly higher compared to M2 macrophages (Fig. 3a, b). We confirmed that osteosarcoma cells treated with CD47 mAb alone did not show significant cell death compared to control IgG-treated cells (*p* > 0.05; Supplementary Fig. S3). This suggested that CD47 mAb had no direct effect on cancer cell viability in the absence of macrophages.

Our flow cytometry data confirmed that M1 macrophages (CD11b/F4/80/CD80^+^) demonstrated increased phagocytic effects (threefold) in the presence of CD47 mAb as compared to control mAb (Fig. 3c). When applying a macrophage-negative gate (CD11b^−^/F4/80^−^) to exclude phagocytic tumor cells, we found 8% baseline tumor cell death in the presence of control antibody and 20% tumor cell death in the presence of CD47 mAb (Supplementary Fig. S2). However, when we gated on total tumor cells, we found 60% tumor cell death in the presence of CD47 mAb as compared to 15% tumor cell death in the presence of control antibody (Supplementary Fig. S2). This suggested that a major portion of CD47 mAb-mediated tumor cell death was a result of M1 macrophage-mediated phagocytosis. M2 macrophages (CD11b/F4/80/CD206^+^) demonstrated very little increase in tumor phagocytic activity in the presence of CD47 antibody (Fig. 3d).

We next examined whether the tumor cells died first and were subsequently phagocytosed or vice versa. We observed tumor cell death during macrophage-mediated phagocytosis with previously established time-lapse confocal microscopy protocols^18^. We found multiple viable tumor cells, which had higher mitochondrial membrane potential at the time of engulfment by M1 macrophages, but showed reduced mitochondrial membrane potential and loss of cell viability after M1 macrophage phagocytosis (Fig. 3e, f). Taken together, these results suggest that CD47 mAb activates M1 macrophages to phagocytose viable cancer cells and that the majority of tumor cell death occurs after phagocytosis.

### CD47 mAb-treated tumors show enhanced ferumoxytol signal on MRI

**Fig. 4:**
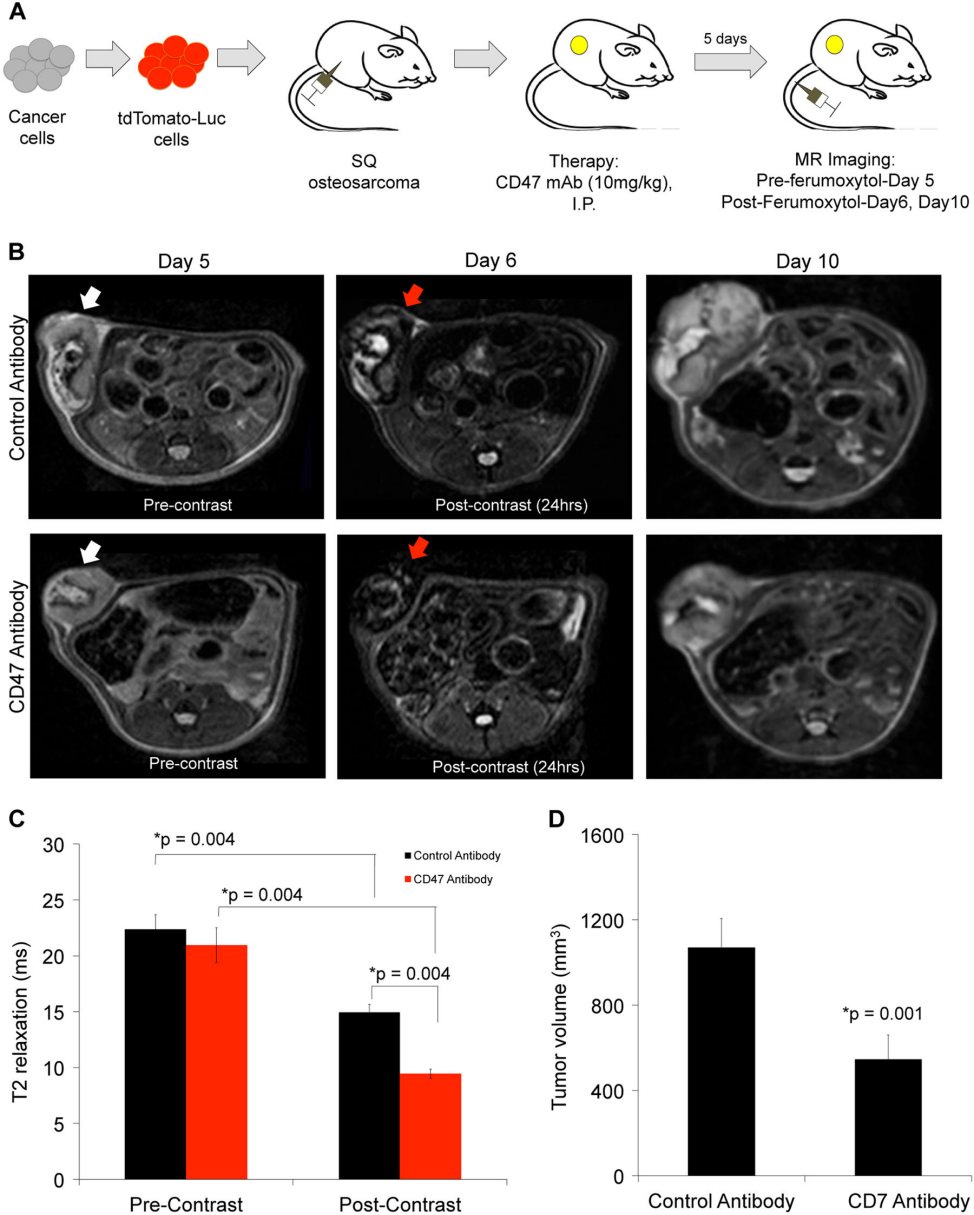
CD47 monoclonal antibody (mAb)-treated tumors show increased ferumoxytol-magnetic resonance imaging (MRI) enhancement. **a** Schematic representation of experimental design. Tumors were initiated in NSG mice (*n* = 6/group) by subcutaneous injection of human MNNG/HOS osteosarcoma cells positive for tdTomato-Luciferase expression. Treatment with control and anti-CD47 mAbs (10 mg/kg, 3× for 5 days) was initiated once the tumors were detected with bioluminescent imaging. MRI was performed on day 5, day 6, and day 10 of therapy. **b** Representative T2-weighted MR images of MNNG/HOS subcutaneous tumors in mice treated with control or CD47 mAbs. The tumors are hyperintense (bright) on pre-contrast MR images (white arrows) and show hypointense (dark) enhancement after ferumoxytol administration (red arrows). **c** Tumor MRI enhancement, quantified as T2 relaxation times, of MNNG/HOS tumors treated with control IgG or CD47 mAbs. CD47 mAb-treated tumors demonstrated significantly shortened T2 relaxation times compared to control antibody-treated tumors on ferumoxytol-enhanced MRI images. **d** Tumor volumes, as measured on T2-weighted MR scans, were significantly smaller on day 10 after anti-CD47 mAb treatment compared to controls. All results are represented as mean ± SD from six tumors per experimental group, *p* value as indicated, exact two-sided Wilcoxon rank-sum tests

**Fig. 5:**
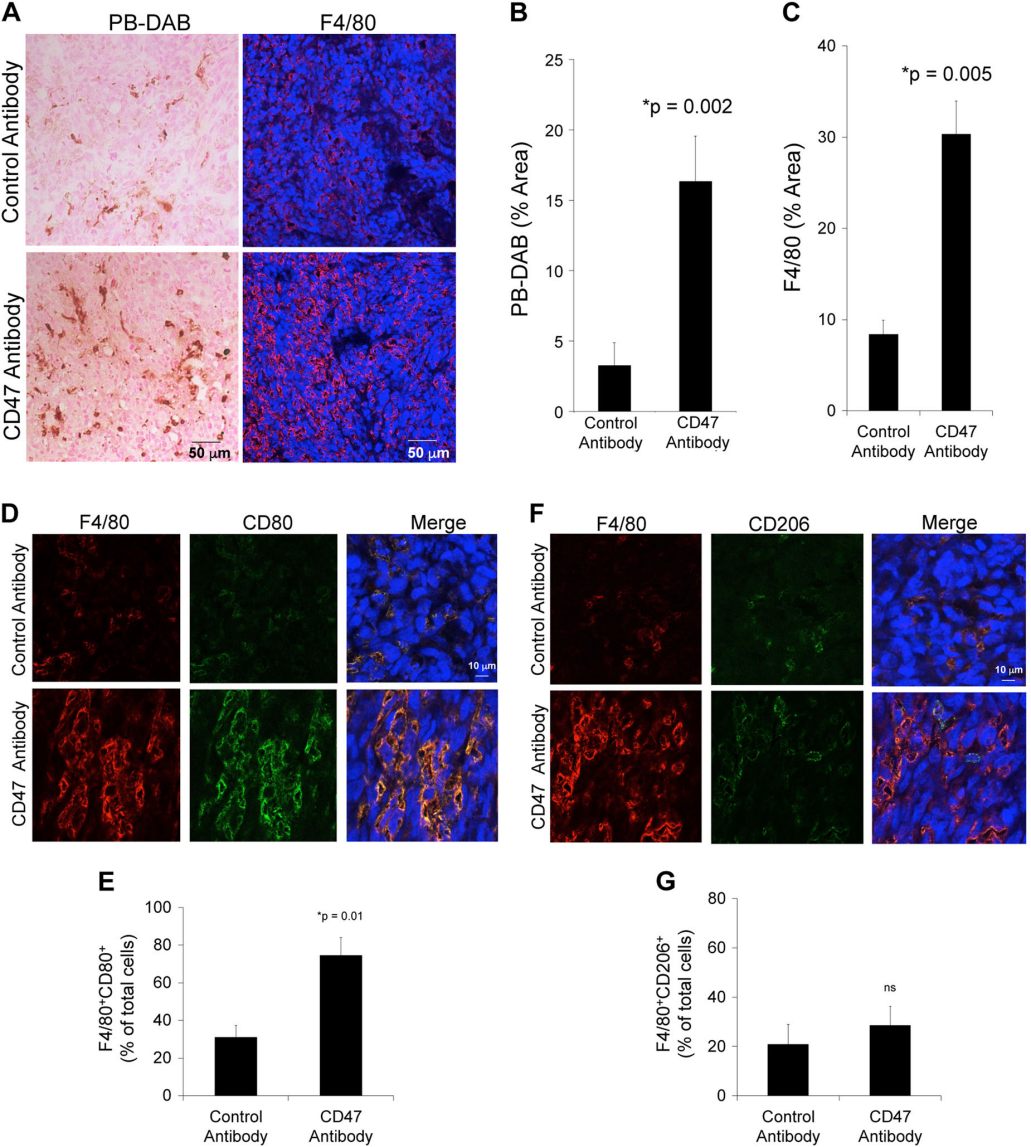
CD47 monoclonal antibody (mAb)-treated tumors show increased ferumoxytol and M1 tumor-associated macrophage (TAM) staining on histopathology. **a** Representative Prussian blue–3,3′-diaminobenzidine (DAB) (scale bar 50 μm) and F4/80 immunofluorescent confocal microscopy images (scale bar 50 μm) of MNNG/HOS tumors depicting iron and F4/80 macrophage staining in response to IgG or CD47 mAb therapy. Corresponding quantitative area of **b** Prussian blue-DAB and **c** F4/80 macrophage staining in control and CD47 mAb-treated tumors. **d**, **f** Immunofluorescent confocal images of F4/80^+^ and CD80^+^ M1 tumor associated macrophages and F4/80^+^ and CD206^+^ M2 tumor associated macrophages (TAM) in control and CD47 mAb-treated MNNG/HOS tumors (scale bar 10 μm). **e**, **g** Corresponding relative percentages of F4/80^+^CD80^+^ M1 TAMs and F4/80^+^CD206^+^ M2 TAMs in control and CD47 mAb-treated MNNG/HOS tumors. All results are represented as mean ± SD from six tumors per experimental group, *p* value as indicated, exact two-sided Wilcoxon rank-sum tests

To investigate whether ferumoxytol-MRI can detect in vivo macrophage response in subcutaneous osteosarcomas, we injected osteosarcoma-bearing mice with ferumoxytol and obtained MR imaging studies before and after CD47 mAb or sham treatment. Subcutaneous MNNG/HOS tumors showed significant hypointense (dark) ferumoxytol enhancement on post-contrast MR scans compared to pre-contrast scans (Fig. 4b). Post-contrast T2 relaxation times of CD47 mAb-treated MNNG/HOS tumors were significantly shorter (1.6-fold, *p* = 0.004) compared to control antibody-treated tumors (Fig. 4c). CD47 mAb-treated tumors were significantly smaller compared to control IgG-treated tumors on day 10 of therapy, indicating successful tumor growth inhibition (*p* = 0.001, Fig. 4d). Iron oxide nanoparticle accumulation in tumors was confirmed by Prussian blue–3,3′-diaminobenzidine (DAB) iron staining: CD47 mAb-treated tumors showed a significantly higher staining score compared to sham-treated tumors (*p* = 0.002, Fig. 5a, b). In addition, CD47 mAb-treated tumors showed evidence of increased TAM quantities and TAM activation: F4/80+ TAM staining (*p* = 0.005, Fig. 5a, c) were significantly higher in CD47 mAb-treated tumors as compared to control antibody-treated tumors. CD47 mAb-treated tumors showed a significantly increased percentage of tumoricidal F4/80/CD80^+^ M1 macrophages (*p* = 0.01, Fig. 5d, e) compared to pro-tumoral F4/80/CD206^+^ M2 macrophages (Fig. 5f, g). M1 polarization of CD47 mAb-treated tumors was also confirmed with flow cytometric analysis (Supplementary Fig. S4).

**Fig. 6:**
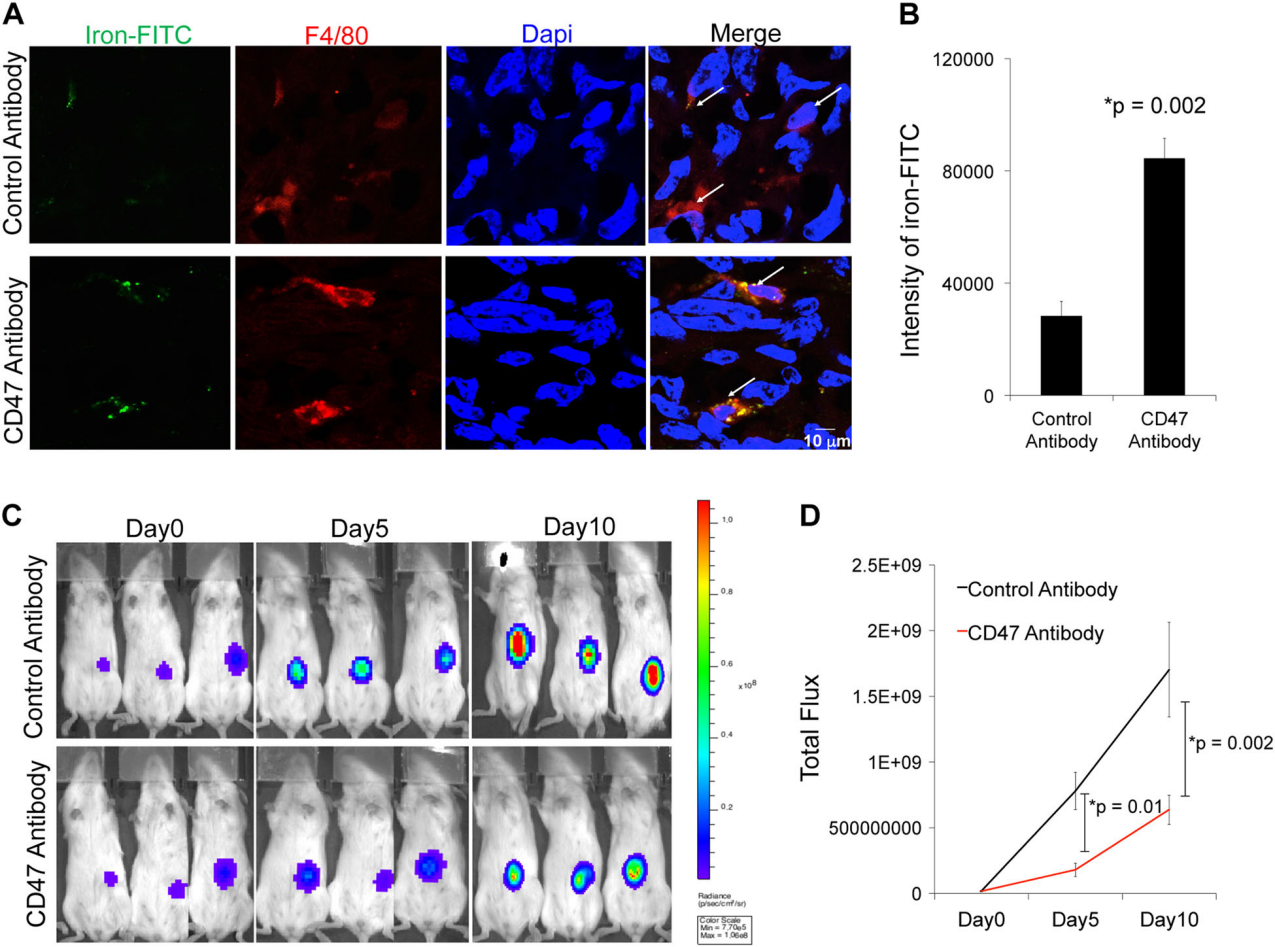
CD47 monoclonal antibody (mAb)-treated mice show increased ferumoxytol uptake by tumor-associated macrophages (TAMs) and reduced tumor burden. **a** Immunofluorescent confocal images of fluorescein isothiocyanate (FITC)-loaded ferumoxytol nanoparticles in F4/80^+^TAMs in MNNG/HOS tumors treated with IgG or CD47 mAb therapy (scale bar 10 μm). **b** Corresponding quantitative signal intensity of iron-FITC per macrophage in control and CD47 mAb-treated tumors. **c** Bioluminescent in vivo images (BLI) of MNNG/HOS tumors in mice treated with control IgG or CD47 mAbs. **d** Bioluminescent signal, quantified as total flux, of control or CD47 mAb-treated tumors on days 0, 5, and 10 of treatment. All results are represented as mean ± SD from six tumors per experimental group, *p* value as indicated, exact two-sided Wilcoxon rank-sum tests

To confirm that the observed tumor enhancement on ferumoxytol-enhanced MR images was due to ferumoxytol nanoparticle compartmentalization in TAM, we injected osteosarcoma-bearing mice with fluorescein isothiocyanate (FITC)-tagged ferumoxytol and obtained fluorescence microscopy images after CD47 mAb or sham treatment: FITC-conjugated ferumoxytol was found in F4/80+ TAMs of all tumors and the corresponding intensity of FITC-ferumoxytol per tumor and per macrophage was significantly higher in CD47 mAb-treated tumors compared to sham-treated controls (*p* = 0.002, Fig. 6a, b). CD47 mAb-treated MNNG/HOS tumors showed significantly reduced tumor flux (*p* = 0.002, Fig. 6c, d) compared to control antibody-treated tumors.

All experiments were repeated in a second tumor model. Similar results were observed in a subcutaneous human osteosarcoma U-2 OS tumor model (Supplementary Fig. S5).

### Ferumoxytol-MRI detects increased TAM in intratibial osteosarcomas after CD47 mAb therapy

**Fig. 7:**
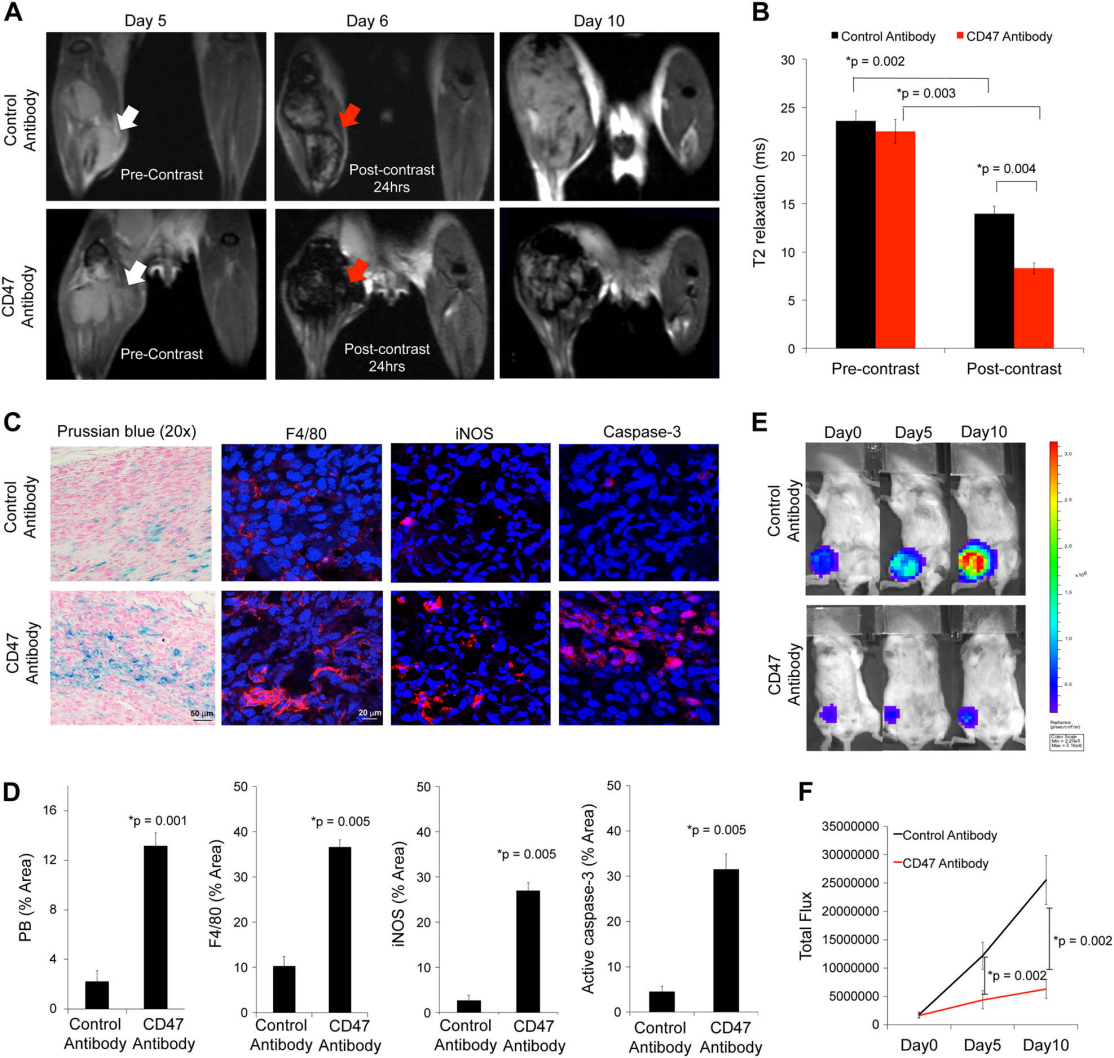
Ferumoxytol-magnetic resonance imaging (MRI) detects tumor-associated macrophage (TAM) response in intratibial syngeneic osteosarcomas after CD47 monoclonal antibody (mAb) therapy. **a** Representative coronal T2-weighted MR images of intratibial K7M2 osteosarcomas at different time points after therapy with control or CD47 mAbs. White arrow points ^tumors^ on pre-contrast images, and red arrow points on post-contrast images. **b** T2 relaxation times of K7M2 osteosarcomas on unenhanced and ferumoxytol-enhanced MRI images at day 6 after control or CD47 mAb therapy. Only ferumoxytol-enhanced MR images show a significantly different T2 relaxation time between CD47 mAb-treated tumors and controls. **c** Prussian blue iron stains and F4/80, CD80, inducible nitric oxide synthase (iNOS) TAM stains, and active caspase-3 stain of K7M2 intratibial tumors treated with control and CD47 mAb. **d** Corresponding quantitative area of Prussian blue iron staining and F4/80, CD80, iNOS TAM staining, and caspase-3 staining in control and CD47 mAb-treated sets. **e** Bioluminescent in vivo images of mice with intratibial K7M2 osteosarcomas before and after therapy with IgG or CD47 mAb. **f** Total quantified flux in control and treated mice with K7M2 osteosarcomas. Results are represented as mean ± SD from six tumors per experimental group, *p* value as indicated, exact two-sided Wilcoxon rank-sum tests

**Fig. 8:**
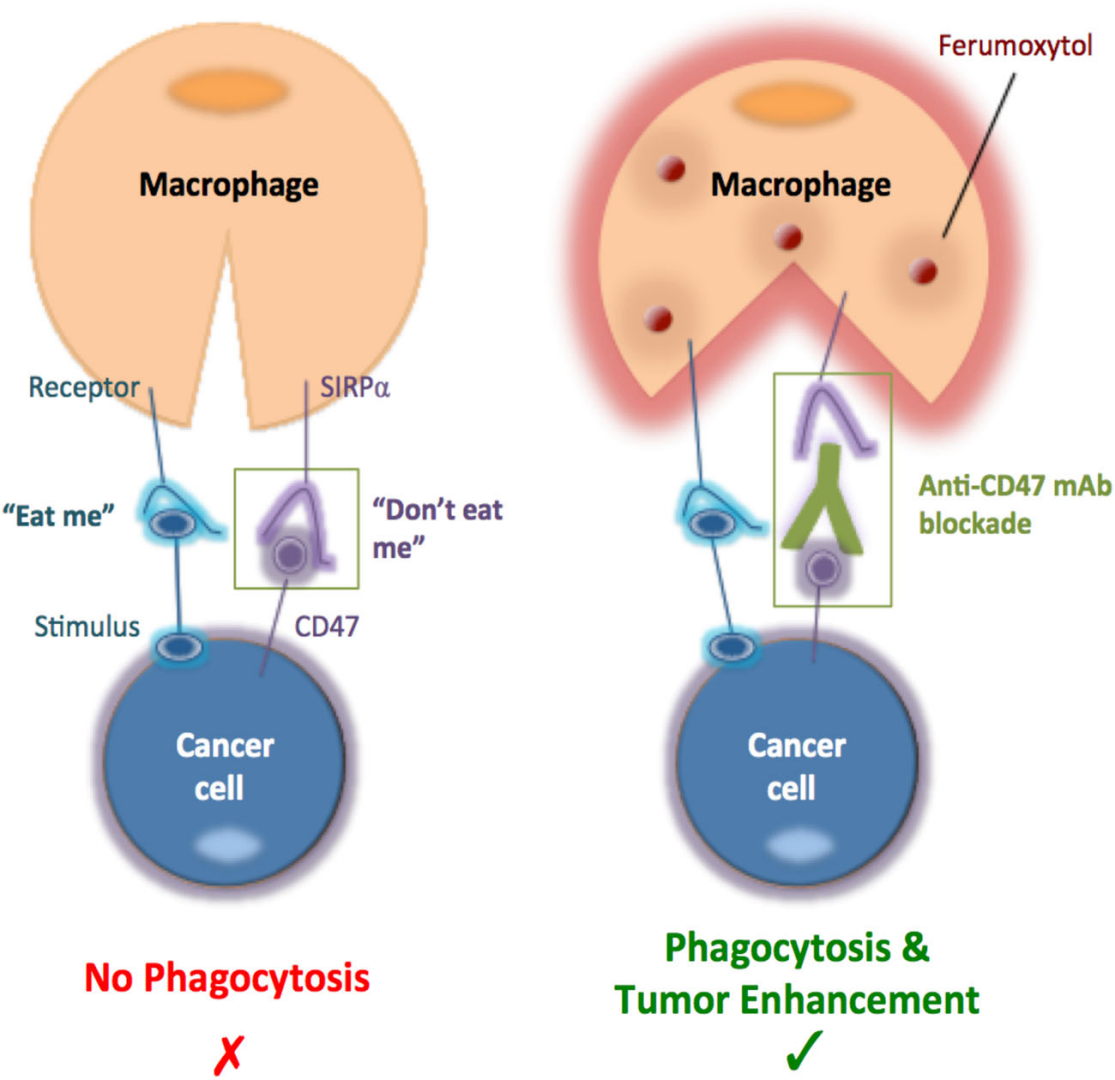
Ferumoxytol-magnetic resonance imaging (MRI) can be an imaging biomarker for CD47 immunotherapy. The signal-regulatory protein alpha (SIRPα) surface marker on osteosarcoma cells inhibits macrophage phagocytosis. Blocking the CD47–SIRPα interaction with CD47 monoclonal antibody (mAb) induces phagocytosis of both tumor and ferumoxytol nanoparticles. Increased uptake of ferumoxytol nanoparticles generates T2 contrast on MR scans that can serve as an imaging biomarker for monitoring responses to CD47 immunotherapy

To investigate whether ferumoxytol-MRI can detect changes in TAM quantities in orthotopic osteosarcomas, we injected osteosarcoma-bearing mice with ferumoxytol and obtained MR imaging studies before and after CD47 mAb or sham treatment. All intratibial K7M2 osteosarcomas showed a hypointense (dark) enhancement on 24 h post-contrast T2-weighted MR images. CD47 mAb-treated tumors showed significantly shorter T2 relaxation times compared to sham-treated tumors (*p* = 0.004, Fig. 7a, b). Compared to pre-contrast scans, CD47 mAb-treated K7M2 tumors showed a threefold decrease in T2 relaxation times on post-contrast MR scans (*p* = 0.003, Fig. 7b), while sham-treated control tumors showed a 1.7-fold reduction in T2 relaxation times (*p* = 0.002, Fig. 7b). CD47 mAb-treated tumors demonstrated significantly increased nanoparticle retention on Prussian blue staining (*p* = 0.001, Fig. 7c, d) and significantly increased levels of F4/80^+^, CD80^+^, iNOS^+^ TAMs (*p* = 0.005, Fig. 7c, d) as compared to control tumors. CD47 mAb-treated K7M2 tumors demonstrated significantly increased active caspase-3 (*p* = 0.005, Fig. 7c, d) and reduced bioluminescence flux (*p* = 0.002, Fig. 7e, f). Taken together, we conclude that ferumoxytol-MRI can monitor tumor response to CD47 mAb therapy non-invasively (Fig. 8).

## Discussion

Our data showed that ferumoxytol-enhanced MRI can detect CD47 mAb-mediated changes in TAM quantities and phagocytic activity in mouse models of osteosarcomas. This provides an important proof of concept for the integration of ferumoxytol-MRI into ongoing clinical trials, which test the efficacy of CD47 mAb for the treatment of solid tumors in patients. Since tumors typically do not decline in size in the immediate post-treatment phase after cancer immunotherapy, our imaging test could fill an important diagnostic gap by providing information about tumor therapy response and overall outcomes.

Our observation of CD47 mAb-mediated TAM activation in osteosarcomas are in accordance with previous studies of Weiskopf et al.^19,20^ in small cell lung cancers, which showed that CD47 mAbs induce TAM influx in tumors and activate TAM phagocytosis. Macrophages recognize tumor cells based on specific surface characteristics, such as exposure of phosphatidylserine and calreticulin ligands on their surface membrane^21,22^. Loss of CD47 surface markers on cancer cells induced macrophage phagocytosis^23^. Confirming this process, we found that CD47 mAb triggered macrophage phagocytosis and tumoricidal effects in osteosarcomas and reduced tumor burden in vivo. Our experiments showed that the majority of cancer cells were phagocytosed alive and subsequently died in macrophages. However, we also found a smaller number of tumor cells that died first and were secondarily phagocytosed. Our experiments showed no direct tumoricidal effect of CD47 mAb. Liu et al.^24^ recently described that most of the anti-tumor effect mediated by the CD47 blockade is specifically CD8^+^ cytotoxic T-cell dependent^25^. CD47 blockade enhances the engulfment of tumor cells by antigen-presenting cells (APCs), enabling the processing and presentation of tumor antigens as peptides in the groove of major histocompatibility complex (MHC) class I molecules for subsequent cross-priming and CD8 T-cell activation.

There is currently no clinically available imaging test which can monitor macrophage response to CD47 mAb in patients. Gadolinium-based contrast agents are the most commonly used MRI contrast agents in the clinic^26^. However, small molecular gadolinium chelates provide non-specific tumor enhancement which does not provide information about the macrophage response in tumors^27,28^. In addition, recent concerns about gadolinium deposition in the brain have led to a search for alternative contrast agents^16,29^. Although other studies have explored radiotracers for TAM detection with positron emission tomography (PET)^30,31^, this modality involves ionizing radiation exposure, which limits its use in children and young adults, the typical patient population with osteosarcomas. Zheleznyak et al.^32^ investigated the utility of CD47 imaging using PET in both human xenograft and murine allograft tumor models. While these studies provide important mechanistic insights, they would be difficult to translate to routine clinical practice. The established clinical imaging modality for local staging and restaging of osteosarcomas to date is MRI, which is more cost effective and more widely available compared to radiotracer-based imaging techniques.

MRI has been used to image macrophages in other pathologies, such as multiple sclerosis^33^, autoimmune encephalomyelitis^34^, infectious knee synovitis^35,36^, and atherosclerotic plaques^37^. Our own team previously established the concept of imaging TAM with ferumoxytol-MRI in mouse models of adenocarcinomas^12^ and brain cancer^38^ as well as in patients with lymphomas and sarcomas^13^. However, nobody investigated, thus far, if this new imaging test can detect changes in TAM quantities due to CD47 mAb therapy. CD47 mAb-treated tumor sections had significantly increased amount of ferumoxytol nanoparticles, which corresponded to ferumoxytol-MRI signals. CD47 mAb activates TAMs and increases TAM-mediated phagocytosis of ferumoxytol nanoparticles, which generates enhanced T2 contrast on MR scans. In addition to being non-invasive and allowing for repeated interrogations of the TAM response in tumors, ferumoxytol-MRI has the distinct advantage over biopsies that it can provide information about the TAM distribution in the whole tumor and thereby avoid sampling errors. This is important, considering the marked heterogeneity of osteosarcomas^39^. Our results provide a novel, immediately translatable and more effective imaging strategy to further interrogate CD47 mAb treatment effects. We showed for the first time that ferumoxytol-enhanced MRI is a reliable biomarker to monitor tumor response and predict outcomes of CD47 mAb immunotherapy. It would be also possible to generate more specific nanoparticles that could target M1 TAM or M2 TAM only^40^. However, such modified nanoparticles would not be clinically applicable.

In conclusion, our study introduces a new and immediately clinically translatable imaging biomarker for TAM response in osteosarcomas to CD47 mAb therapy. This could represent a significant breakthrough for clinicians as a new gold-standard imaging test for treatment stratification and tracking TAM response in upcoming CD47 mAb immunotherapy trials of osteosarcomas and other solid tumors.

## Materials and methods

### Chemicals and antibodies

The following antibodies were used: Alexa Fluor 647 goat anti-mouse IgG (Invitrogen), NL557 conjugated goat anti-rat IgG (R&D), anti-mouse F4/80 (R&D Biosystems), anti-mouse MMR/CD206 (R&D Biosystems), anti-mouse B7-1/CD80 (R&D Biosystems), anti-mouse iNOS (Abcam), anti-mouse active caspase-3 (Abcam), PE/Cy7 anti-human CD47 (BioLegend, clone CC2C6), anti-dextran FITC (StemCell Technologies, clone DX1), APC/Cy7 anti-mouse LY-6G/Ly-6C (Gr-1) (BioLegend, clone RB6-8C5), PE/Cy7 anti-mouse CD45 (BD Bioscience, clone 30-F11), PerCP/Cy5.5 anti-mouse/human Cd11b (Biolegend, clone M1/70), APC anti-mouse F4/80 (eBioscience, clone BM8), Brilliant Violet 785™ anti-mouse I-A/I-E (MHC-II) (BioLegend, clone M5/114.15.2), Brilliant Violet 605™ anti-mouse CD86 (BioLegend, clone GL-1), PE anti-mouse CD206 (BioLegend, clone C068C2), PE/Cy7 anti-mouse CD124 (IL-4R alpha) (BioLegend, clone I015F8), Alexa Fluor® 488 anti-mouse CD80 (BioLegend, clone 16-10A1), CD163 (SantaCruz Biotechnology, M-96), CD68/SR-D1 (R&D Biosystems, clone #298807), anti-human CD47 (BioXcell, clone B6.H12), anti-mouse CD47 (BioXcell, clone MIAP301), CD47 (R&D Biosystems), human IgG control, mouse IgG1 isotype control (BioXcell), and anti-mouse CD16/CD32 (Mouse BD Fc Block™) (BD Biosciences, Clone 2.4G2). The following chemicals were used: Ferumoxytol (Feraheme™, AMAG Pharmaceuticals), Prolong™ Gold anti-fade reagent with DAPI (Invitrogen), Zombie Aqua™ Fixable Viability Kit (BioLegend), Cellbrite™ red/green (Biotium), cisplatin (Sigma), and oxaliplatin (Sigma).

### CD47 gene expression analysis in human osteosarcoma

We evaluated the expression of CD47 in de-identified osteosarcoma specimen from eight chemotherapy-naive human patients, one de-identified osteoma specimen, one de-identified normal bone specimen (Cooperative Human Tissue Network), three human osteosarcoma cell lines (U-2 OS, Saos-2, and MNNG/HOS: ATCC) and a normal bone cell line (hFOB 1.19: ATCC). Assessment of CD47 gene expression in these samples was performed by qPCR as previously described^38,41^, using glyceraldehyde 3-phosphate dehydrogenase (GAPDH) as a control marker. CD47, CD68, and CD163 protein level in these samples was evaluated by immunofluorescence on optimal cutting temperature (OCT) compound-embedded, fresh-frozen tissue as described previously^14^.

### In vitro evaluation of the therapeutic efficacy of CD47 mAb against osteosarcoma cells

In vitro studies were performed in three human osteosarcoma cell lines (Saos-2, U-2 OS, MNNG/HOS, ATCC, Manassas, VA, USA), and one murine osteosarcoma cell line (K7M2, ATCC, Manassas, VA, USA). Saos-2, U-2 OS, and K7M2 cells were cultured in Dulbecco’s modified Eagle's medium (Life Technologies) supplemented with 10% fetal bovine serum (FBS), 100 units/mL of penicillin, and 100 mg/mL of streptomycin. MNNG/HOS cells were grown in Eagle's minimum essential medium (ATCC) supplemented with 10% FBS, 100 units/mL of penicillin, and 100 mg/mL of streptomycin. A normal bone cell line hFOB 1.19, ATCC was used as a negative control. All cell lines used were authentic and confirmed to be mycoplasma negative using the MycoAlert Mycoplasma Activity kit (Lonza).

The following antibodies were used for treatment purpose: anti-human CD47 (BioXcell, Clone B6.H12), anti-mouse CD47 (BioXcell, clone MIAP301), human IgG control, and mouse IgG1 isotype control (BioXcell).

To evaluate macrophage-mediated tumor phagocytosis^42^ in the presence of CD47 mAb, Saos-2 osteosarcoma cells were labeled with 1,1′-Dioctadecyl-3,3,3,’,3′-tetramethylindodicarbocyanine (CellBrite™ Green, Biotium) according to the manufacturer’s protocol, and incubated at a 1:1 ratio with bone marrow-derived mouse macrophages in serum-free (Iscove's modified Dulbecco's medium (IMDM), with or without 10 μg/mL CD47 mAb at 37 °C for 4 h.

To evaluate uptake of iron nanoparticles by macrophages in the presence of CD47 mAbs, we co-cultured murine bone marrow derived macrophages with unlabeled MNNG/HOS cells and FITC-labeled ferumoxytol (0.01 mM) with or without 10 μg/mL CD47 mAb at 37 °C for 4 h.

Macrophages were then stained with F4/80 antibody and subsequently imaged using Leica SP8 confocal microscopy. Tumor phagocytosis was calculated as the percentage of macrophages positive for phagocytized CellBrite™ Green^+^ cells. FITC-ferumoxytol signal in F4/80^+^macrophage was quantified to demonstrate nanoparticle uptake by macrophages.

Flow cytometry assay was used to confirm macrophage-mediated phagocytosis of osteosarcoma cells and macrophage-mediated tumoricidal effects. For this CellBrite™ Green-labeled MNNG/HOS cells were co-cultured with murine M1 and M2 macrophages as described above. Fluorescently labeled antibodies targeting macrophage markers (CD11b,F4/80, CD80, and C206) were used to identify the M1 and M2 macrophage population. Phagocytosis was quantified by the percentage of CellBrite™ Green events among CD11b+F4/80+ macrophage events. Tumor cell death in macrophage co-cultures was assessed with Aqua amine cell viability staining in the CellBrite™ Green+ population.

Time-lapse tumor phagocytosis and cell death of MNNG/HOS osteosarcoma cells caused by M1 macrophages in the presence of CD47 mAb was performed to identify whether tumor cell death preceded phagocytosis or vice versa. Osteosarcoma cells were labeled with JC-1 mitochondrial membrane dye and M1 macrophages were stained with Hoechst33342, and co-incubated at a 1:1 ratio with CD47 mAb or control mAb and time-lapse imaged every 180 s for 4 h using LSM 880 Airyscan confocal microscope using Zen Black software at 63× objectives. In viable cells with normal, polarized mitochondria, JC-1 shows red J-aggregate fluorescence (590 nm), but upon losing viability as mitochondrial potential reduces, the red J-aggregate fluorescence changes to cytoplasmic diffusion of green monomer fluorescence (530 nm). Intensity of JC-I fluorescence at 590 nm and 530 nm for 100 tumor cells was calculated using velocity software. Ratio of JC-1 fluorescence at 590 and 530 was used to represent mitochondrial membrane potential at different time points during phagocytosis.

For preparation of mouse macrophages, 7–11-week-old NSG (NOD.Cg-*Prkdc*^*scid*^ *Il2rg*^*tm1Wjl*^/SzJ) mice were euthanized and the *femora* and *tibiae* were isolated. The bones were kept in ice-cold phosphate buffered saline (PBS) and sterilized in 70% ethanol. By flushing them with mouse macrophage medium (IMDM with 10% FBS, 1× penicillin/streptomycin, 200 mM glutamine, and 25 mM HEPES, all from Corning Inc.), bone marrow cells were gathered and plated at 1 × 10^6^/ml in Petri dishes in mouse macrophage medium. To generate macrophages, bone marrow cells were treated for 7 days with recombinant mouse macrophage colony-stimulating factor (25 ng/mL, Shenandoah Biotech). For polarization of M1 and M2 macrophages^42^, murine bone marrow cells were treated for 7 days with either recombinant human or mouse macrophage colony-stimulating factor (M-CSF; 25 ng/mL). M2 polarization was achieved by further treatment on days 5 and 6 with IL-4 (20 ng/mL) and IL-13 (20 ng/mL). To generate M1 macrophages, bone marrow cells were treated for 7 days with either recombinant human or mouse granulocyte macrophage colony-stimulating factor (GM-CSF; 25 ng/mL). M1 polarization was achieved with further treatment on day 5 by interferon-γ (20 ng/mL) stimulation for 1 h, followed by lipopolysaccharide for 48 h (100 ng/mL; Sigma-Aldrich). Unless otherwise stated, all cytokines were purchased from Shenandoah Biotech.

### In vivo evaluation of the therapeutic efficacy of CD47 mAb

All animal maintenance, handling, surveillance, and animal procedures were performed in accordance with and approval from the Stanford University Administrative Panel on Laboratory Animal Care (Protocol 24965).

Twenty-four anesthetized 6–8‐week‐old NSG mice received subcutaneous injections of 2 × 10^6^ MNNG/HOS or U-2 OS tumor cells in matrigel^14^. MNNG/HOS or U-2 OS tumor cells were engineered to express luciferase-td Tomato as described previously^43^. For subcutaneous implantation, cells were suspended in Matrigel® and 100 μL of cell suspension (2 × 10^6^ cells) was injected subcutaneously under the ventral skin of anesthetized 6–8‐week‐old NSG mice^14^. Tumors were treated with CD47 mAb (clone B6H12, 10 mg/kg) or isotype IgG1 control Ab (*n* = 6/group) by intraperitoneal injection three times for 5 days.

Twelve anesthetized mice received intratibial injections of 5 × 10^5^ K7M2 tumor cells. K7M2 tumors were treated with CD47 mAb (clone MIAP301, 10 mg/kg, *n* = 6) or isotype control antibodies (*n* = 6) by intraperitoneal injections three times for 5 days.

### MR imaging

To monitor ferumoxytol tumor enhancement all mice underwent MRI after 5 days of CD47 mAb therapy or control IgG (sham) treatment, using a 7T MR scanner (Bruker-Agilent Technologies-General Electric Healthcare). All mice received a pre-ferumoxytol scan on day 5 and a post-ferumoxytol scan on day 6 and day 10 after CD47 mAb therapy or sham treatment. The following pulse sequences were used before and after intravenous injection of ferumoxytol (Feraheme™, AMAG Pharmaceuticals, 30 mg/kg): T2 fast spin echo (FSE) sequences with a repetition time (TR) of 4500 ms, an echo time (TE) of 42 ms, and a flip angle α: 90° and T2-weighted multi-slice multi-echo (MSME) sequences with a TR of 3000 ms, a TE of 8, 16, 24, 32, 40, 48, 56, 64, 72, 80, 88, and 96 ms and α: 90°; a field of view of 2 cm × 2 cm and a slice thickness of 0.5 mm for the MRI acquisitions. T2 relaxation times of the whole tumor were measured with Osirix software. Region of interest (ROI) was drawn around tumor on T2 maps generated by Bruker software and T2 relaxation times were computed with Osirix software. To calculate tumor volumes the area of tumor ROIs on T2 FSE scans (day 10) were first summed up and then multiplied by slice thickness^38^.

Twelve additional mice with MNNG/HOS subcutaneous tumors were injected with FITC-loaded ferumoxytol nanoparticles to correlate MRI findings with immunohistopathology.

### Bioluminescent imaging

Tumor growth over time was monitored using bioluminescent imaging (BLI) on days 0, 5, and 10 on an IVIS Spectrum (Caliper Life Science) as described previously^38^. Luminescent imaging was performed on an IVIS Spectrum (Caliper Life Science) and quantified using Living Image 4.0 software. D-luciferin (firefly) potassium salt solution (Biosynth) was prepared (15 mg/mL) and injected intraperitoneally (0.139 g luciferin per kg body weight). Total flux (photons per second) values were obtained by imaging mice until peak radiance was achieved and quantified with Living Image 4.0 software^14,38^.

### Immunocytochemistry

After completion of all imaging procedures, tumors were explanted, fixed, sectioned, and stained with Prussian blue iron staining and F4/80, CD80, CD206, inducible nitric oxide synthase (iNOS) and active caspase-3 immunofluorescence staining as described previously^14^. CD47, CD68, and CD163 immunofluorescence staining on cell lines and patient specimens were performed as described previously. For Prussian blue iron stains, tissue sections of formalin-fixed, paraffin-embedded tissue were deparaffinized with xylene, rehydrated, and stained according to the manufacturer’s recommendation with the Sigma-Aldrich Accustain Iron Stain Kit. DAB-Quanto kit (Thermo Scientific) was used to generate Prussian blue-DAB stains. Sections were counterstained with nuclear fast red (Fisher Scientific). Representative images were captured using AxioImager Widefield Fluorescence Microscope with a 20× objective for whole-slide imaging. For immunofluorescence staining, samples were permeabilized with 0.1% Triton X-100 in PBS for 10 min, washed 3 × 5 min in PBS, and blocked with 3% bovine serum albumin (BSA) in PBS for 30 min. Samples were then incubated with primary antibodies overnight at 4 °C before being washed 3× in PBS for 5 min each. Secondary antibodies were added for 2 h in the dark and washed 3 × 5 min in PBS, before samples were mounted with 4′,6-diamidino-2-phenylindole (DAPI) mounting media (Invitrogen). Immunofluorescence images were acquired with a Leica SP8 confocal microscope using Leica AF software at 20× and 40× objectives. Images were analyzed using ImageJ and Velocity software. The percent area covered by Prussian Blue and F4/80-positive macrophages in tumor xenografts was quantitated using freely available ImageJ software as described previously^13^. The percent area covered by CD47, CD68, and CD163 in five osteosarcoma patient specimens was quantitated over four high-power fields using freely available ImageJ. The number of F4/80+CD80+ and F4/80+CD206+ macrophages in tumor xenografts was quantitated using ImageJ software and expressed as percentage of total cells. Signal intensity of CD47 staining in cancer cell lines and iron-FITC staining in F4/80-positive macrophages was measured with velocity software.

### Flow cytometry

For immunophenotyping macrophages, control and anti-CD47 mAb-treated mice were killed, and subcutaneous tumors were dissociated to single cells and stained with APC/Cy7 anti-mouse LY-6G/Ly-6C (Gr-1) (BioLegend), PE/Cy7 anti-mouse CD45 (BD Bioscience), PerCP/Cy5.5 anti-mouse/human Cd11b (Biolegend), APC anti-mouse F4/80 (eBioscience), Brilliant Violet 785™ anti-mouse I-A/I-E (MHC-II) (BioLegend), Brilliant Violet 605™ anti-mouse CD86 (BioLegend), PE anti-mouse CD206 (BioLegend), PE/Cy7 anti-mouse CD124 (IL-4R alpha) (BioLegend), and Alexa Fluor® 488 anti-mouse CD80 (BioLegend), all according to the manufacturers’ specifications. Endogenous mouse FcγII and FcγII receptors were blocked using 3% BSA containing Mouse BD Fc Block^TM^ (1 μg). Flow cytometric analysis was performed on a BD FACS ARIA II (BD) flow cytometer. Data analysis was performed using FlowJo Version 9.6.4 (Tree Star, Ashland, OR, USA). Live singlets were gated using FSC-W/FSC-H. Gates were drawn using Fluorescent Minus One control tubes.

### Statistical analysis

Differences in CD47 mRNA for osteosarcoma, osteoma, and normal bone samples were tested by exact two-sided Wilcoxon rank-sum tests. Differences in CD47, CD68, and CD163 in these samples were tested by exact two-sided Wilcoxon rank-sum tests. Results from in vitro experiments and results from in vivo tumor T2 relaxation time and BLI total flux measurements were compared between experimental groups using exact two-sided Wilcoxon rank-sum tests.. Statistical analyses were performed using GraphPad Prism (GraphPad, San Diego, CA, USA) and Stata Release 15 (StataCorp LP, College Station, TX) software. The level of significance was set at *p* *<* 0.05 for all analyses.

## Supplementary information

Supplementary Figure Legends Clean Copy

Supplementary figure S1. CD47 expression in osteosarcoma cell lines

Supplementary figure S2. CD47 inhibition triggers macrophage-mediated tumor cell phagocytosis and tumor cell death in vitro

Supplementary figure S3. CD47 mAb does not induce any direct tumoricidal effects in vitro

Supplementary figure S4. Flowcytometric analysis of M1 polarization in osteosarcomas treated with CD47 mAb

Supplementary figure S5. Ferumoxytol-MRI of U-2 OS subcutaneous tumors after CD47 mAb
