# Supplementary material for: Nanoparticle enhanced MRI can monitor macrophage response to CD47 mAb immunotherapy in osteosarcoma
Source: Cell Death Dis. 2019 Jan 15;10(2):36. doi: 10.1038/s41419-018-1285-3 (PMC6367456; doi:10.1038/s41419-018-1285-3)
Supplement: Supplementary file 1 — Supplementary Figure Legends Clean Copy [file 41419_2018_1285_MOESM1_ESM.docx]

**Supplementary Figure Legends**

**Supplementary figure S1.** **CD47 expression in osteosarcoma cell lines: (A)** Representative immunofluorescence images of CD47 staining in murine osteosarcoma cell line K7M2 and human osteosarcoma cell lines MNNG/HOS and Saos-2. Tumor cells stained with CD47 antibody show positive staining compared to cells stained with isotype control antibody, (scale bar, 25 μm). **(B)** Corresponding quantification of CD47 staining in tumor cells. Results are represented as mean ± SD from five independent experiments, p value as indicated, exact two-sided Wilcoxon ranksum tests.

**Supplementary figure S2. CD47 inhibition triggers macrophage-mediated tumor cell phagocytosis and tumor cell death *in vitro*:** MNNG/HOS tumor cells were co-cultured with murine M1 macrophages in presence of control and CD47 mAb. Gating strategy to identify total tumor cells (cellbrite green^+^), tumor cells pre phagocytosis (F4/80^-^/CD11b^-^) and tumor cells post phagocytosis (F4/80^+^/CD11b^+^/cellbrite green^+^). Flowcytometry contour plots showing total tumor cell death (upper panel), phagocytosis-mediated tumor cell death (middle panel) and phagocytosis-independent tumor cell death (lower panel) in **(A)** control and **(B)** CD47mAb-treated sets. Corresponding charts showing percent total tumor cell death **(C),** percent phagocytosis **(D)** and percent tumor cell death pre-phagocytosis **(E).** Data are displayed as means ± SD of five experiments per group, p value as indicated, exact two-sided Wilcoxon ranksum tests.

**Supplementary figure S3. CD47 mAb does not induce any direct tumoricidal effects *in vitro*.** Chart showing percent tumor cell death in MNNG/HOS cells incubated directly with control and CD47mAb for 24 hrs. Data are displayed as means ± SD of three experiments per group, p value as indicated, exact two-sided Wilcoxon ranksum tests.

**Supplementary figure S4. Flowcytometric analysis of M1 polarization in osteosarcomas treated with CD47 mAb. (A)** Flow cytometry dot plots show gating strategy for identifying CD45+GR1-CD11b+F4/80+ TAMs in control and CD47 mAb treated MNNG/HOS subcutaneous tumors. **(B)** Representative histograms of CD80 and IL-4Rα protein expression in CD45+GR1-CD11b+F4/80+ gated TAMs from control and CD47 mAb treated MNNG/HOS subcutaneous tumors. **(C)** Chart shows percentages of M1 and M2 TAMs in control and CD47 mAb treated MNNG/HOS subcutaneous tumors. All results are represented as mean ± SD from six tumors per experimental group, p-value as indicated, exact two-sided Wilcoxon ranksum tests.

**Supplementary figure S5.** **Ferumoxytol-MRI of U-2 OS subcutaneous tumors after CD47 mAb. (A)** Representative axial T2-weighted MR images of U-2 OS subcutaneous tumors in mice treated with control IgG or CD47 mAb. MRI was performed on day 5 and day 6 of therapy. The CD47 mAb treated tumor shows stronger ferumoxytol enhancement as stronger dark (negative) enhancement on T2-weighted MR images (red arrows). **(B)** Tumor MRI enhancement, quantified as T2 relaxation times, of U-2 OS tumors treated with control IgG or CD47 mAbs. CD47 mAb-treated tumors demonstrated significantly shortened T2 relaxation times compared to control antibody-treated tumors on ferumoxytol-enhanced MRI images. **(C)** Subcutaneous U-2 OS osteosarcomas showed less luminescence after CD47 mAb therapy (10 mg/kg, 3x/week) compared to control tumors treated with IgG. **(D)** Bioluminescent signal, quantified as total flux, of CD47 mAb treated tumors (red) was significantly lower compared to IgG treated control tumors. All results are represented as mean ± SD from six tumors per experimental group, exact two-sided Wilcoxon ranksum tests.
