# Supplementary figures and images for: Nanoparticle enhanced MRI can monitor macrophage response to CD47 mAb immunotherapy in osteosarcoma
Source: Cell Death Dis. 2019 Jan 15;10(2):36. doi: 10.1038/s41419-018-1285-3 (PMC6367456; doi:10.1038/s41419-018-1285-3)

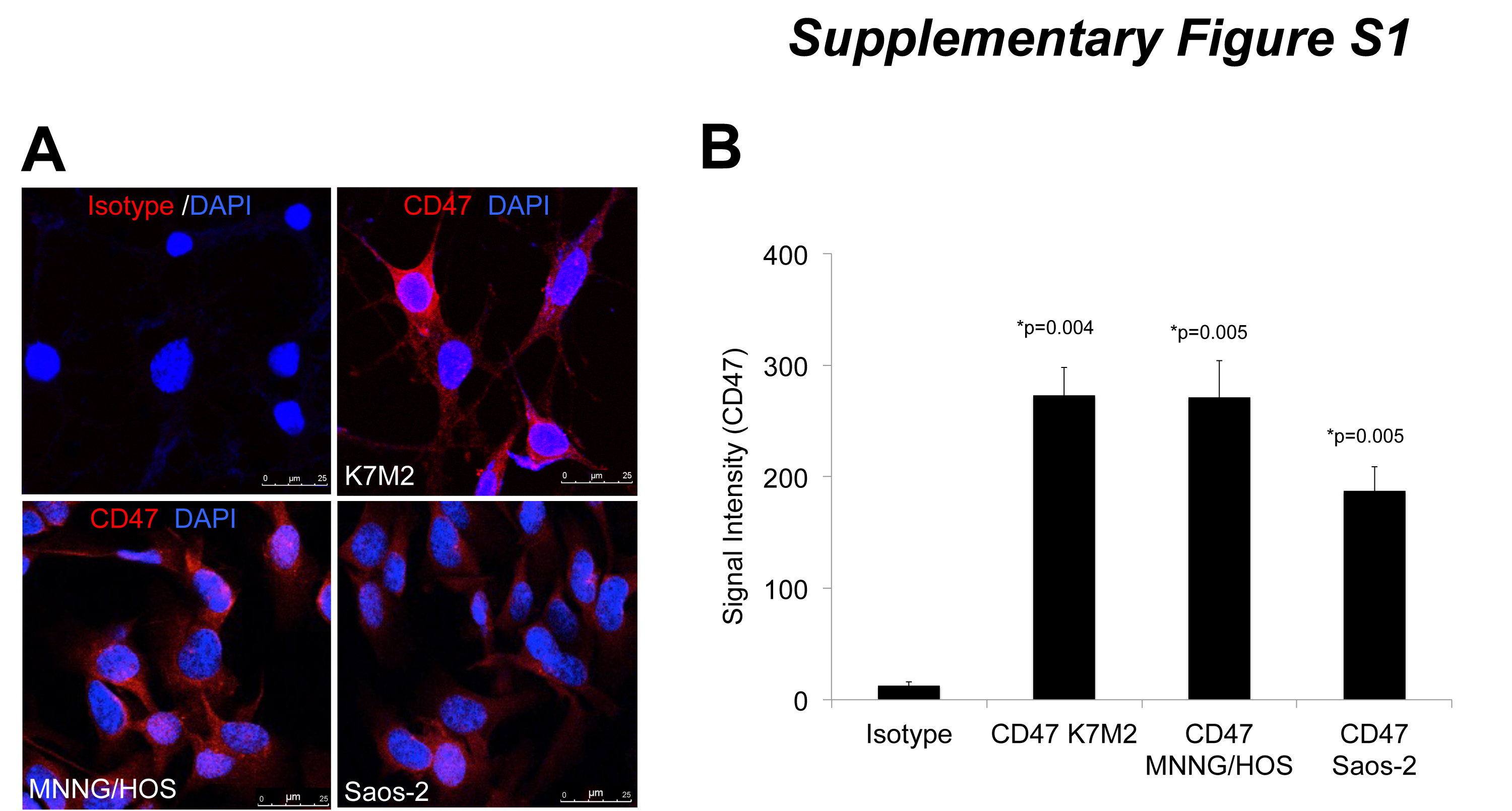

Supplement: Supplementary file 2 — Supplementary figure S1. CD47 expression in osteosarcoma cell lines [file 41419_2018_1285_MOESM2_ESM.jpg]

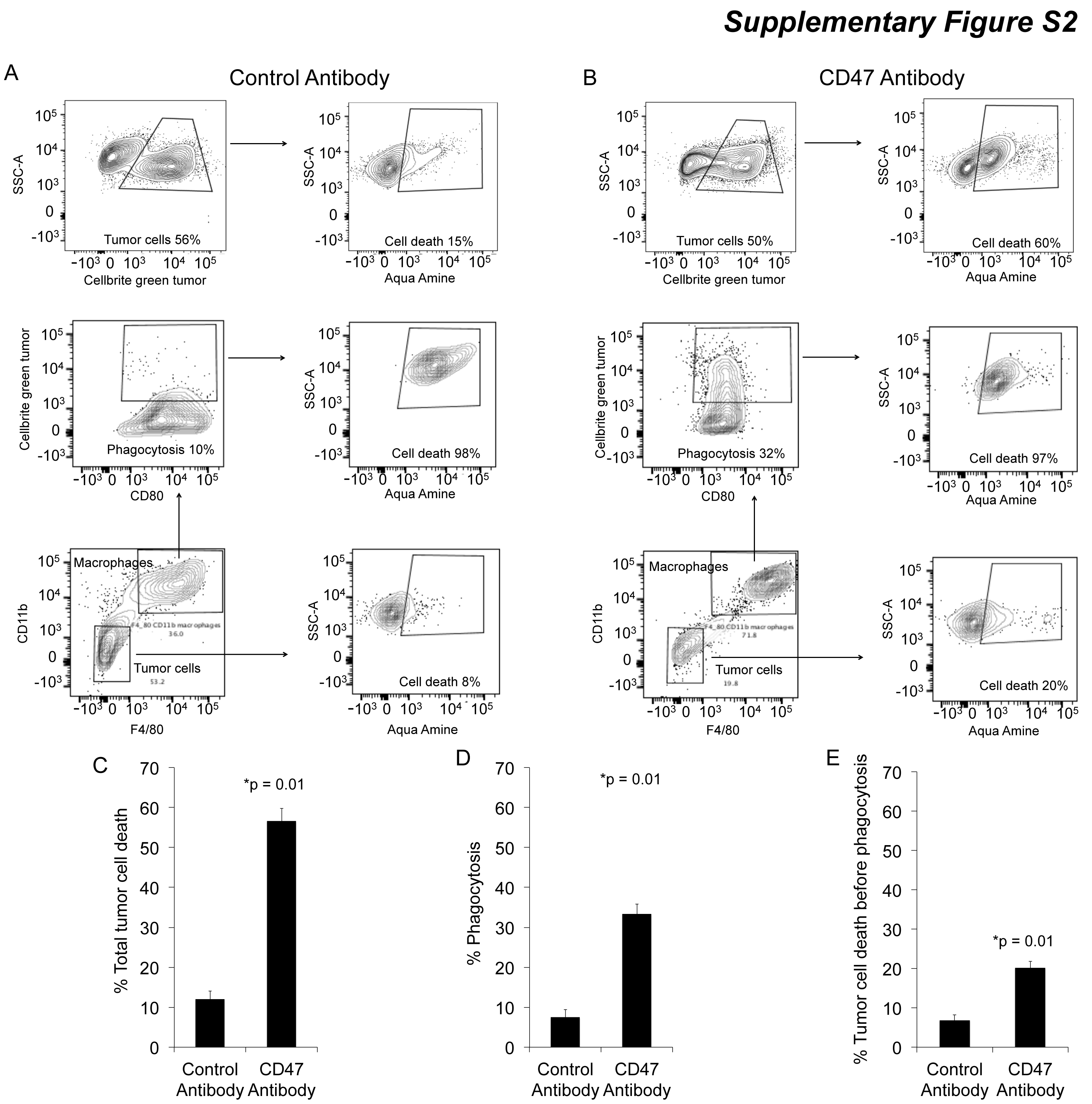

Supplement: Supplementary file 3 — Supplementary figure S2. CD47 inhibition triggers macrophage-mediated tumor cell phagocytosis and tumor cell death in vitro [file 41419_2018_1285_MOESM3_ESM.jpg]

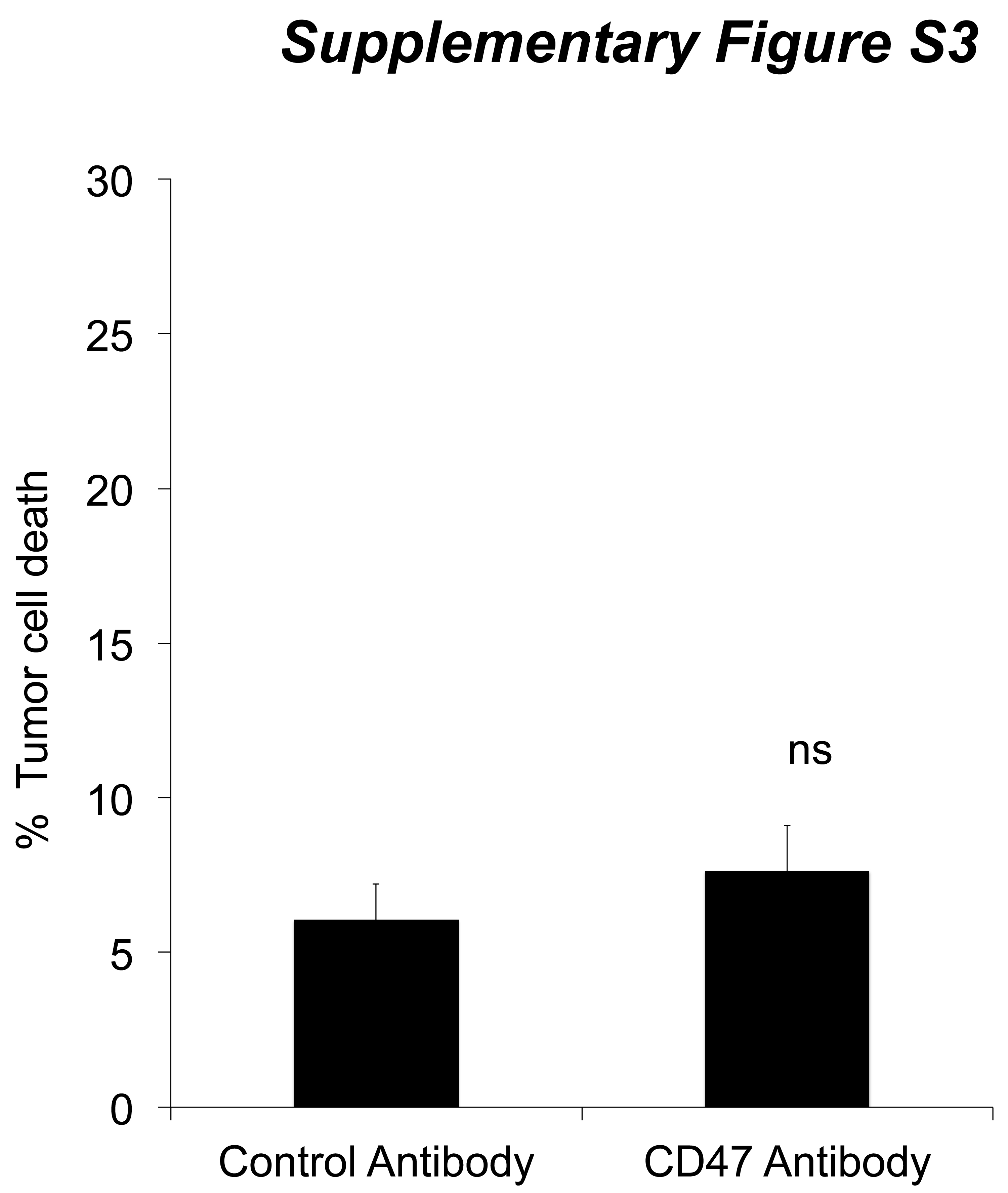

Supplement: Supplementary file 4 — Supplementary figure S3. CD47 mAb does not induce any direct tumoricidal effects in vitro [file 41419_2018_1285_MOESM4_ESM.jpg]

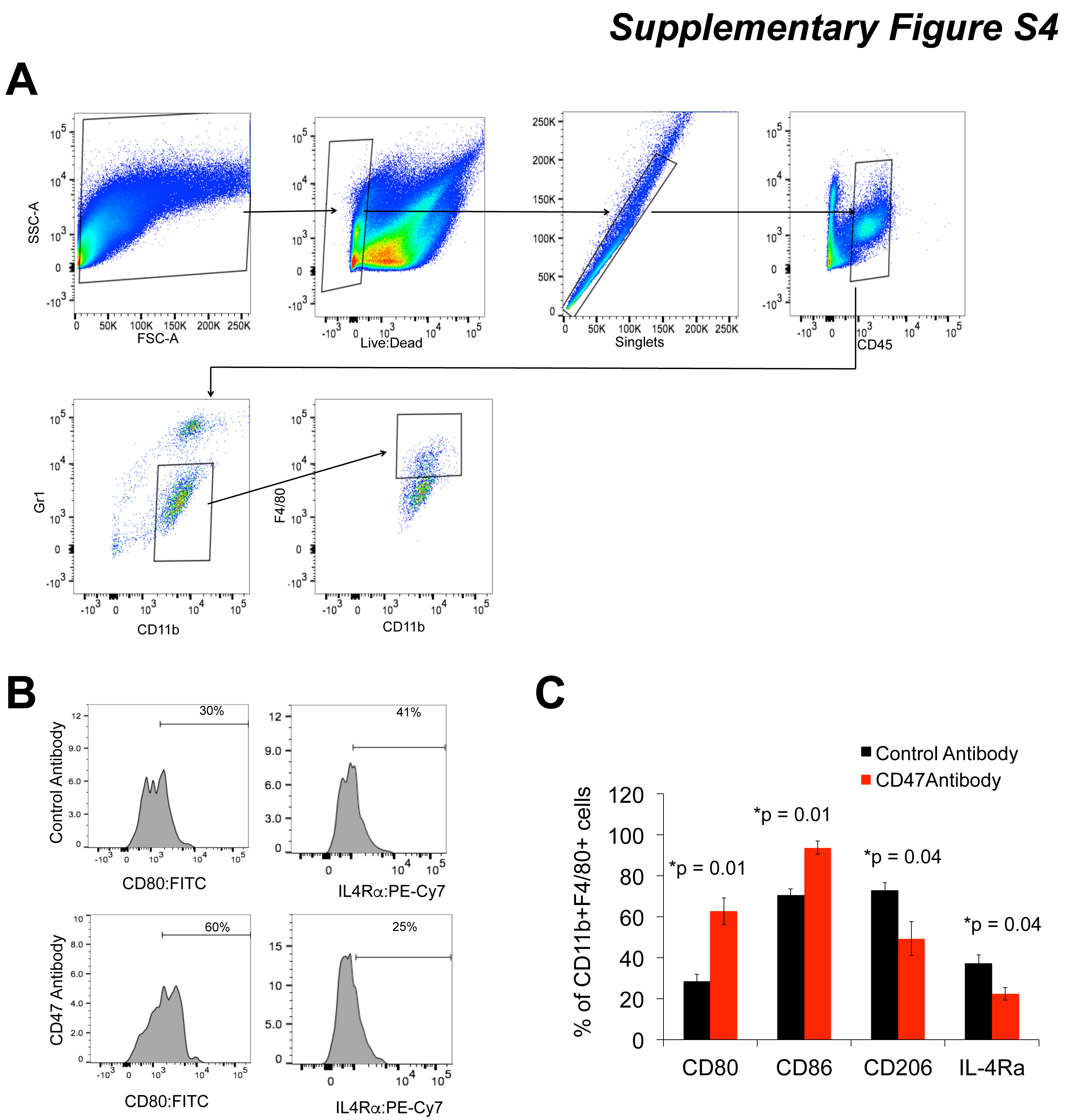

Supplement: Supplementary file 5 — Supplementary figure S4. Flowcytometric analysis of M1 polarization in osteosarcomas treated with CD47 mAb [file 41419_2018_1285_MOESM5_ESM.jpg]

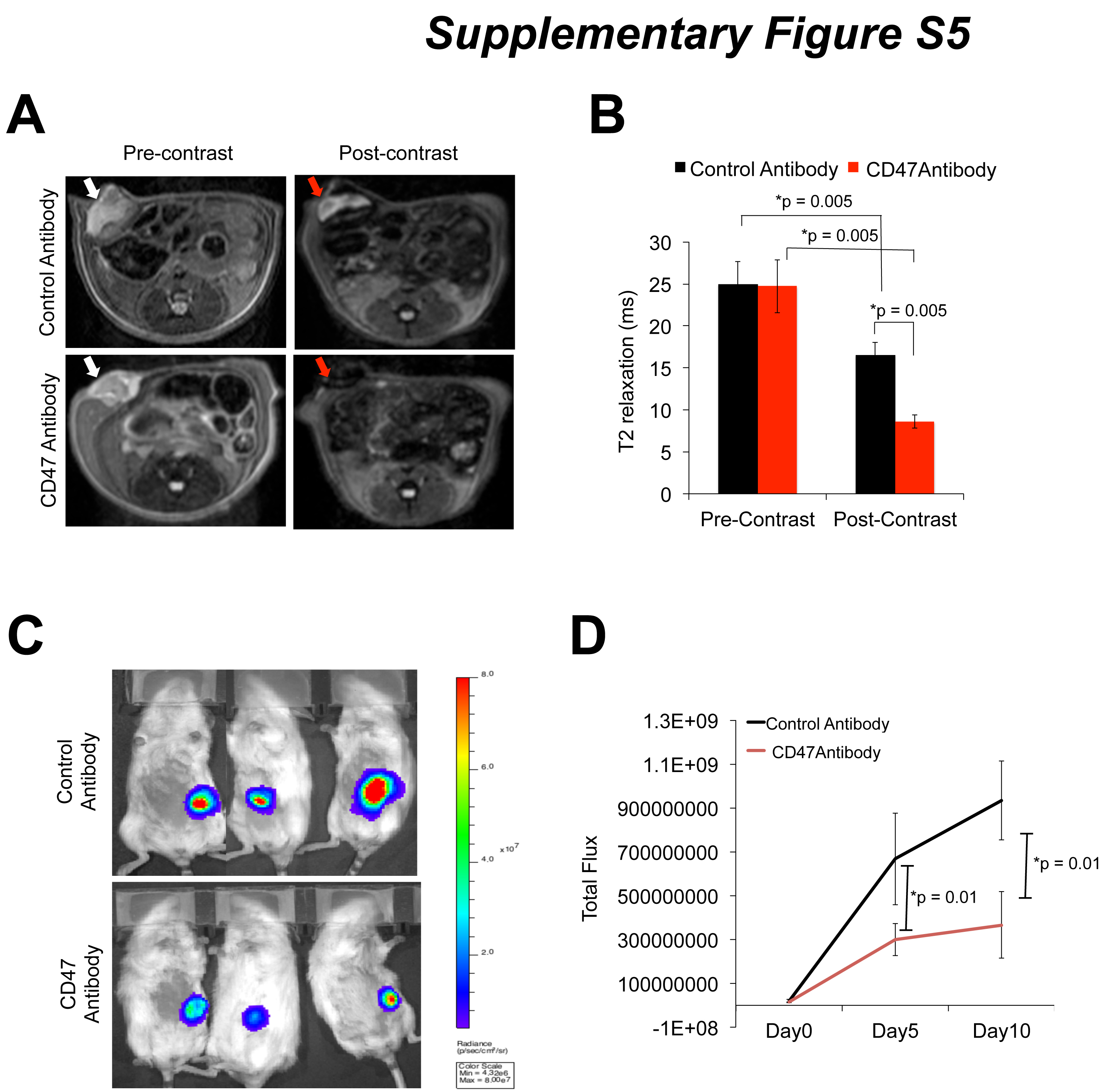

Supplement: Supplementary file 6 — Supplementary figure S5. Ferumoxytol-MRI of U-2 OS subcutaneous tumors after CD47 mAb [file 41419_2018_1285_MOESM6_ESM.jpg]
